# Whole-genome sequencing and phylogenetic analysis capture the emergence of a multi-drug resistant *Salmonella enterica* serovar Infantis clone from diagnostic animal samples in the United States

**DOI:** 10.3389/fmicb.2023.1166908

**Published:** 2023-06-02

**Authors:** Mariela E. Srednik, Brenda R. Morningstar-Shaw, Jessica A. Hicks, Christopher Tong, Tonya A. Mackie, Linda K. Schlater

**Affiliations:** ^1^National Veterinary Services Laboratories, Animal and Plant Health Inspection Service, U.S. Department of Agriculture, Ames, IA, United States; ^2^Center for Veterinary Biologics, Animal and Plant Health Inspection Service, U.S. Department of Agriculture, Ames, IA, United States

**Keywords:** *Salmonella* Infantis, pESI- like, megaplasmids, extended spectrum beta-lactamases, whole genome sequencing, phylogeny, antimicrobial resistance, integron (intI1 and intI2)

## Abstract

**Introduction:**

*Salmonella enterica* is a major cause of foodborne illness in the United States. A multi-drug resistant (MDR) emergent *Salmonella* Infantis (ESI) with a megaplasmid (pESI) was first identified in Israel and Italy and subsequently reported worldwide. The ESI clone carrying an extended spectrum β-lactamase *bla*CTX-M-65 on a pESI-like plasmid and a mutation in the *gyr*A gene has recently been found in the United States in poultry meat.

**Methods:**

We analyzed the phenotypic and genotypic antimicrobial resistance, genomics and phylogeny of 200 *S. infantis* isolates from animal diagnostic samples.

**Results:**

Of these, 33.5% were resistant to at least one antimicrobial and 19.5% were multi-drug resistant (MDR). Eleven isolates from different animal sources were phenotypically and genetically similar to the ESI clone. These isolates had a D87Y mutation in the *gyr*A gene conferring reduced susceptibility to ciprofloxacin and harbored a combination of 6–10 resistance genes: *bla*CTX-M-65, *aac*(3)-IVa, *aad*A1, *aph*(4)-Ia, *aph*(3′)-Ia, *flo*R, *sul*1, *dfr*A14, *tet*A, and *fos*A. These 11 isolates carried class I and class II integrons and three virulence genes: sinH, involved in adhesion and invasion, *ybt*Q and *ybt*P, associated with iron transport. These isolates were also closely related to each other (separated by 7 to 27 SNPs) and phylogenetically related to the ESI clone recently found in the U.S.

**Discussion:**

This dataset captured the emergence of the MDR ESI clone in multiple animal species and the first report of a pESI-like plasmid in isolates from horses in the U.S.

## 1. Introduction

*Salmonella enterica* is a major cause of foodborne illness in the United States. *Salmonella enterica* subspecies *enterica* serovar Infantis has ranked among the top 10 serotypes causing human illness in the United States during the last 10 years and was the 6^th^ most commonly isolated *Salmonella* serotype in the National Enteric Disease Surveillance Annual report in 2016 (Centers for Disease Control and Prevention, 2018). Poultry are most commonly associated with *S. infantis* outbreaks, although the serotype has been found in other food animal sources such as cattle and swine (Tate et al., 2017).

In the United States, the National Antimicrobial Resistance Monitoring System (NARMS) conducts routine surveillance for antimicrobial resistance in *Salmonella* from retail meats. *S. infantis* comprises 2–4% of the surveyed isolates with most being pan-susceptible to antimicrobials on the NARMS panel (Tyson et al., 2021). The Diagnostic Bacteriology and Pathology Laboratory within the National Veterinary Services Laboratories (NVSL) routinely serotypes *Salmonella* isolates submitted by private, state, and federal laboratories as well as veterinarians, researchers, and other animal health officials. During the period of this study, *S. infantis* was ranked number 10 among clinical isolates but below 10 in non-clinical isolates during 2014; whereas in 2017 *S. infantis* was ranked number seven among both clinical and non-clinical isolates submitted for *Salmonella* serotyping in 2017.

In 2018, one of the largest foodborne outbreaks in the U.S. caused by *Salmonella* was linked to raw chicken containing *S. infantis*. There were 129 cases in 32 states. Twenty-five people were hospitalized, and one death was reported in New York (Centers for Disease Control and Prevention, 2019). The outbreak strain was isolated from both live chickens and many types of raw chicken products, suggesting it was likely widespread in the broiler industry. The outbreak strain was resistant to multiple class-representative antimicrobial agents: ampicillin, ceftriaxone, chloramphenicol, ciprofloxacin, fosfomycin, gentamicin, hygromycin, kanamycin, nalidixic acid, streptomycin, sulfamethoxazole, tetracycline, and trimethoprim-sulfamethoxazole.

A multi-drug resistant (MDR) emergent *S. infantis* (ESI) with a large megaplasmid was identified by Aviv et al., in Israel in 2014 (Aviv et al., 2014), and as similar (pESI-like) plasmid by Franco et al. (2015) in Italy in 2015, and subsequently worldwide (Cartelle et al., 2016; Tate et al., 2017; Szmolka et al., 2018; Acar et al., 2019; Bogomazova et al., 2020; Burnett et al., 2021). In Israel, the megaplasmid was described as a chimeric IncI/IncP plasmid and named pESI (Aviv et al., 2014). In Italy, a similar megaplasmid from broilers and broiler meat was identified and named pESI-like (Franco et al., 2015). In the United States, a temporal and geographical study (2014–2019) found a pESI-like clone in retail meats in Tennessee in 2014. By 2019, this plasmid was found throughout the U.S. in *Salmonella* isolated primarily from retail chicken (29%) and turkey (7%) products (Tyson et al., 2021). This plasmid was classified as an IncFIB-type plasmid and assumed to be the result of multiple recombination events (Tyson et al., 2021). In contrast to the implications of this report, there are different clones and clonal groups of ESI reported by Franco et al. (2015), Tate et al. (2017), Szmolka et al. (2018), Nagy et al. (2020), among others. Their comparison and differences in accessory genomes are presented by Mazel (2006).

The efficient dissemination of this *S. infantis* clone may be due to the substantial advantages and fitness of this strain in the environment and host (Aviv et al., 2014). The pESI clone found in Israel and Italy is characterized by a *gyr*A mutation and several antimicrobial resistance genes, often including a CTX-M extended-spectrum β-lactamase (ESBL), heavy metal resistance genes, biocides, and presence of virulence genes (Aviv et al., 2014; Franco et al., 2015).

Rapid spread of genetic information between bacteria is facilitated by horizontal transfer and acquisition of mobile and integrative genetic elements such as plasmids, transposons, and integrons. Integrons are fundamental elements in the emergence of MDR and bacterial evolution (Deng et al., 2015). Integrons can capture antimicrobial resistance gene cassettes and are classified according to the amino acid sequence of the integrase gene (*int*l) that is located at the 5′ conserved site (CS). *Intl*1, *intl*2 and *intl*3 are associated with mobile genetic elements and are found in class 1, class 2 and class 3 integrons, respectively (Rodríguez et al., 2006). Class 1 integrons are the most prevalent in *Salmonella* clinical isolates (U.S. Food and Drug Administration, 2021).

The objective of this study was to analyze phenotypic and genomic antimicrobial resistance and genetic relationships in *S. infantis* isolates from veterinary diagnostic samples recovered between 2014 and 2017 in the United States, and notably we identified and analyzed the emergence of the pESI-like plasmid carrying clone in isolates from these animal sources.

## 2. Materials and methods

### 2.1. Bacterial strains

A total of 200 *S. infantis* isolates representing multiple animal species (swine = 74, cattle = 50, horses = 27, chickens = 24, cats/dogs = 12, turkeys = 9, goats = 2, sheep = 2) from 31 states were selected from diagnostic submissions to the National Veterinary Services Laboratories (NVSL) for *Salmonella* serotyping between the years of 2014 and 2017. The dataset was initially limited to one sample per year per owner. Following selection, isolates were stripped of identifying information other than basic metadata including animal species, year, and state of origin. Diagnostic isolates were stored long term at ambient temperature on nutrient agar slants. Selected isolates were plated on sheep blood agar (Remel, Lenexa, KS) and a single colony was expanded for further testing with a portion frozen for future use. Identity was confirmed via matrix assisted laser desorption/ionization (MALDI-TOF) using an Autoflex Speed® LRF (Bruker Daltonics, Billerica, MA); broth microdilution antimicrobial susceptibility was performed using the Sensititre SWIN System (Thermo Fisher Scientific, Waltham, MA, USA); and nucleic acid was extracted using the Promega Maxwell® RSC 48 Instrument (Madison, WI). Isolates were sequenced with a targeted depth of 40-80X using 2 × 250 paired end chemistry and the NexteraXT library preparation kit (Illumina®). Isolate sequences are publicly available in the National Center for Biotechnology Information (NCBI) BioProject PRJNA789479.

For the phylogenetic analysis, we included the ERR1014119 emergent *S. infantis* strain from Italy (Franco et al., 2015), and 14 U.S. pESI-like isolates from chicken/turkey meat, human illness, and the environment from NCBI, a random selection from diverse branches of the NCBI Pathogens tree representing *S. infantis* isolates (Supplementary Table 1), and the *S. infantis* strain NZ CP016408 as the reference.

### 2.2. Antimicrobial susceptibility testing

All isolates were tested against 14 class-representative antimicrobial agents: ampicillin (AMP), amoxicillin/clavulanic acid (AMC), cefoxitin (FOX), ceftriaxone (CRO), meropenem (MEM), chloramphenicol (CHL), ciprofloxacin (CIP), nalidixic acid (NAL), gentamicin (GEN), streptomycin (STR), sulfisoxazole (FIS), trimethoprim/sulfamethoxazole (SXT), tetracycline (TET) and azithromycin (AZM) using the CMV4AGNF plate and interpreted using criteria established by the NARMS (CLSI, 2020). NARMS breakpoints are adopted from CLSI (Clinical and Laboratory Standards Institute; Bankevich et al., 2012), except for streptomycin and azithromycin, which have no CLSI breakpoints. Isolates with ciprofloxacin MICs categorized as intermediate (MIC 0.12 to 0.5 μ/ml) or resistant (MIC ≥ 0.12 μg/ml) by CLSI are defined as having decreased susceptibility (DS) to CIP (MIC ≥ 0.12 μg/ml) by NARMS as a marker for emerging fluoroquinolone resistance.

### 2.3. *In silico* bioinformatics analysis

Raw data was assembled using Spades (Feldgarden et al., 2021) and assemblies were subjected to further analysis. Isolates were analyzed for the presence of antimicrobial resistance genes and plasmids using AMRfinderPlus (Seemann, 2014), Abricate (Carattoli et al., 2014) with ResFinder and NCBI databases, and PlasmidFinder (Zankari et al., 2017). Analysis criteria included a threshold of identity ≥80% and coverage ≥80% of the target gene. Mutations were identified using PointFinder (Torres-Elizalde et al., 2021). Integrons were identified using the IntFinder 1.0 tool described by Lee et al. (2009). The presence of integrases was determined using NCBI reference sequences of the *intl*1 (MG785026.1), *intl*2 (MK994977.1), and *Intl*3 (KM194584.1) using Geneious Prime v11.0.9 (Biomatters Ltd., NZ).

### 2.4. Phylogenetic analysis

Multilocus Sequence Type (MLST) was determined using Abricate (Carattoli et al., 2014) with the PubMLST databases. Phylogenetic relationship was analyzed with vSNP^1^ using *S. infantis* strain NZ CP016408 as the reference isolate carrying the *bla*_CTX-M-65_ gene from food animal in the U.S. (Tate et al., 2017).

### 2.5. Statistical analysis

A logistic regression comparing the odds ratio, *p*/(1-*p*), where the *p* represents the proportion of isolates with resistance, was used to evaluate the dependence of the ratio on year or on species, using likelihood ratio tests. These calculations were performed using R software version 3.5.3.

## 3. Results

### 3.1. Phylogeny

Ninety-nine percent of the isolates (*n* = 198) were classified as sequence type (ST) 32 by MLST: *aro*C(17), *dna*N(18), *hem*D(22), *his*D(17), *pur*E(5), *suc*A(21), and *thr*A(19). Only two isolates did not identify as an established MLST pattern. One of these isolates (from swine) has a new allele *pur*E(~5) instead of *pur*E(5), and the other (from chicken) has a new combination of alleles *pur*E(460) instead of *pur*(5). Both isolates were pan-susceptible.

**Figure 1:**
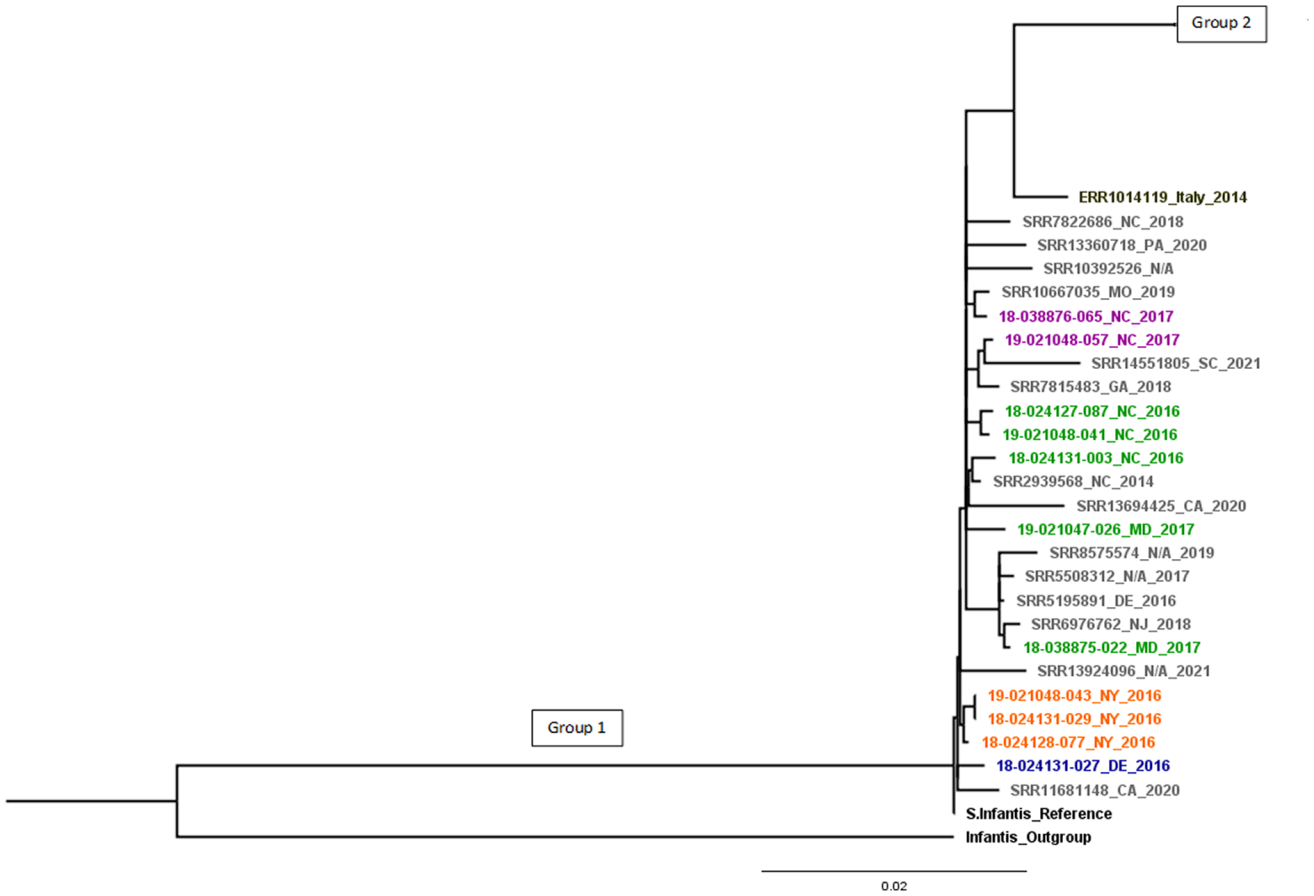
pESI-like cluster. Different animal species are in colors: cattle (blue), horses (orange), chickens (green), turkeys (purple), U.S. pESI-like positive isolates from chicken/turkey meat, human illness, and the environment from NCBI (in grey) and emergent *S. infantis* strain from Italy (in black). Group 1 shows pESI-like positive isolates.

The SNP tree-based phylogenetic analysis (Supplementary Figure 1) shows two distinct phylogenetic branches. Isolates related to the emergent *Salmonella* Infantis (ESI) clone carrying several common resistance genes and the IncFIB-like replicon are found in Group 1, this group contains the 11 diagnostic isolates studied, besides the reference strain. The Group 1 in Figure 1 contains the 11 diagnostic isolates plus 14 U.S. pESI-like positive isolates from NCBI, besides the reference strain. They showed genetic relatedness and were found to derive from a common ancestor (18-024131-027).

Compared to the reference, there were 7 to 283 SNPs between the isolates in Group 1. Group 2 showed differences of 1 to 336 SNPs.

All pESI-like positive isolates were separated by only 7 to 27 SNPs from the most recent common ancestor, indicating that isolates from chicken/turkey meat, human illness, and the environment are closely related to isolates from chickens, turkeys, horses, and cattle. When compared the pESI-like isolate from Italy, this strain is separated by 43 SNPs from Group 1 and 106 SNPs from Group 2 (Figure 1). Within Group 1, smaller groups can be seen that are associated to a common animal source.

*S. infantis* isolates within Group 2 do not show the same antimicrobial pattern and genomic characteristics as Group 1 containing the pESI-like megaplasmid. Group 2 isolates are divided into 13 branches (Supplementary Figure 1).

### 3.2. Antimicrobial resistance genes, point mutations, integrons, and plasmids

Several antimicrobial resistance genes were found (Supplementary Figure 1; Table 1) in the isolates from this study. The *aac*(6′)-laa gene was found in all the isolates. The *tet*A gene (*n* = 25) was the most frequent tetracycline resistance gene, *sul*1 (*n* = 23) was the most frequent sulfonamide resistance gene, *aad*A1 (*n* = 16) the most frequent streptomycin resistance gene, *aac*(3)-IVa (*n* = 15) the most frequent gentamicin-resistance gene, and *bla*_CMY-2_ (*n* = 14) gene was the most frequent β-lactam-resistance gene. We found extended-spectrum β-lactamases (ESBLs) encoded by *bla*_SHV_ (*n* = 2) and *bla*_CTX-M-65_ (*n* = 10) genes. These enzymes provide multiple resistance to β-lactams, including penicillins and many cephalosporins from the first to the fourth generation, apart from cefoxitin. The 2 isolates carrying the *bla*_SHV-12_ and 10 isolates carrying the *bla*_CTX-M-65_ genes showed resistance to ampicillin and ceftriaxone. One isolate carrying the *bla*_CTX-M-65_ gene was phenotypically susceptible to β-lactams. Three macrolide resistance genes (*mph*A, *mph*E, *msr*E) were detected in a single isolate, and in five isolates the *ere*A gene that encodes an erythromycin esterase was detected. Resistance to colistin was not tested, but we found 2.5% (*n* = 5) of isolates carrying the *mcr*-9.1 gene.

**Table 1:** Antimicrobial resistance (AMR) genes, point mutations, replicons, integron classes, phylogeny group, MLST, and other gene elements in *n* = 200 *Salmonella* Infantis isolated from animals.

AMR class	AMR gene (*n*, % of all isolates)
β-lactams	*bla*_CMY-2_ (14, 7.0%), *bla*_TEM-1_ (10, 5.0%), *bla*_CTX-M-65_ (10, 5.0%), *bla*_SHV-12_ (2, 1.0%), *bla*_CMY-130_ (2, 1.0%)
Phenicols	*flo*R (17, 8.5%), *cat*A2 (2, 1.0%), *cml*A (1, 0.5%),
Quinolones	*qnr*B19 (2, 1.0%)
Aminoglycosides	*aac*(6′)-laa (200, 100%), *aph*(3′)-Ia (23, 11.5%), *aad*A1 (16, 8.0%), *aph*(4)-Ia (15, 7.5%), *aph*(3″)-Ib (14, 7.0%), *aph*(6)-Id (14, 7.0%), *aad*A2 (6, 3.0%), *aad*A7 (2, 1.0%), *aac*(3)-IVa (15, 7.5%), *aac*(3)-II (3, 1.5%), *aac*(3)-*VIa* (3, 1.5%), *aac*(6′)-IIc (3, 1.5%), *ant*(2″)-la (2, 1.0%), *aac*(3)-IId (1, 0.5%)
Folate pathway antagonists	*sul*1 (23, 11.5%), *sul*2 (13, 6.5%), *sul*3 (1, 0.5%), *dfr*A14 (10, 5.0%), *drf*A19 (2, 1.0%),
Tetracyclines	*tet*A (25, 12.5%), *tet*B (13, 6.5%), *tet*D (3, 1.5%), *tet*M (1, 0.5%),
Macrolides	*ere*A (5, 2.5%), *mph*A (1, 0.5%), *mph*E (1, 0.5%), *msr*E (1, 0.5%)
Phosphonic acids (fosfomycin)	*fos*A3 (7, 3.5%), *fos*A354827590 (1, 0.5%)
Ansamycins (rifamycin)	*arr* 269927220 (3, 1.5%)
Polymyxins (colistin)	*mcr*-9.1 (5, 2.5%)
Glycopeptides (bleomycin)	*ble*O (1, 0.5%)
AMR class	Point mutations (*n*, % of all isolates)
Quinolones	*gyr*A(D87Y) (11, 5.5%), *gyr*A(S83Y) (2, 1.0%)
Plasmids	Replicon (*n* % of all isolates)
	Col440I (6, 0.03%), Col8282 (1, 0.005%), ColRNAI (1, 0.005%), IncA/C2 (10, 0.05%), IncFIB(K)_1_Kpn3 (11, 0.055%), IncFIB(pB171)_1_pB171 (1, 0.005%), IncHI2A (9, 0.045%), IncHI2 (9, 0.045%), ncI1_1_Alpha (25, 0.125%), IncL/M (1, 0.005%), IncP1_2 (1, 0.005%), IncP1_3 (1, 0.005%), IncQ1 (1, 0.005%), IncX1 (2, 0.01%), IncY (4, 0.02%), RepA_1_pKPC-CAV1321 (9, 0.045%), p0111 (2, 0.01%)
Integrons	Integrase (*n*, % of all isolates)
Class I	*Intl*1 (26, 0.13%)
Class II	*Intl*2 (11, 0.055%)
Phylogeny	Group (*n* % of all isolates)
	Group 1 “pESI clone” (11, 0.055%), Group 2 (189, 0.95%)
MLST	ST (*n* % of all isolates)
	ST32 (198, 99%), no established MLST (2, 0.001%)
Other elements	Gene (*n* %)
Multidrug and metal efflux complex	*mds*A (200, 100%), *mds*B (200, 100%)
Biocides	*qac*Δ1 (23, 11.5%), *qac*E (0.5%), *qac*L (0.5%)
Virulence	*sin*H (200, 100%), *iro*B (200, 100%), *iro*C (200, 100%), *ybt*Q (11, 5.5%), *ybt*P (11, 5.5%)
Heavy metals	*ars*R (200, 100%)*, ars*C, (3 1.5%), *gol*S (200, 100%), *gol*T (200, 100%), *mer*A 17 (8.5%), *mer*P (20, 10.0%), *mer*B (9, 4.5%), *mer*T (31, 15.5%), *mer*R (31, 15.5%), *mer*C (14, 7.0%), *mer*E (16, 8.0%), *mer*D (16, 8.0%), *sil*E (10, 5.0%), *sil*S (10, 5.0%), *sil*R (10, 5.0%), *sil*C (10, 5.0%), *sil*F (10, 5.0%), *sil*B (10, 5.0%), *sil*A (10, 5.0%), *sil*P (10, 5.0%), *pco*E (8, 4.0%), *pco*D (6, 3.0%), *pco*R (6, 3.0%), *pco*S (10, 5.0%), *pco*A (4, 2.0%), *pco*B (4, 2.0%), *pco*C (4, 2.0%), *ter*W (9, 4.5%), *ter*Z (9, 4.5%), *ter*D (9, 4.5%)
Heat protein	*shs*P (2, 1%)

A *gyr*A point mutation in D87Y was found in 5.5% (*n* = 11), and S83Y in 1.0% (*n* = 2) of isolates. Isolates presenting the D87Y point mutation were related to the pESI-like clone.

In addition, some isolates harbored biocide, virulence, and stress response genes (Supplementary Figure 1). All isolates harbored the *mds*A/B genes encoding a membrane fusion protein of the multidrug and metal efflux complex MdsABC, the virulence genes *sin*H, *iro*B and *iro*C, and the *ars*R, *gol*S, *gol*T genes, involved in heavy metal resistance.

Seventeen plasmid types were identified in 30.5% (*n* = 61) of isolates with the most common being Incl1-Alfa (12.5%, *n* = 25), IncFIB(K)-1-kpn3 (5.5%, *n* = 11), and IncA/C2 (5%, *n* = 10). Isolates positive for the *mcr*-9.1 gene related to colistin resistance and/or *arr*-269,927,220 gene related to rifamycin resistance had 3 plasmids in common: IncHI2A, IncHI2 and RepA-1-pKPC-CAV1321.

Using the IntFinder 1.0 tool, we identified 23 class 1 integrons and 9 class 2 integrons (Table 2). We found the presence of class 1 integrons in 13% (*n* = 26), and class 2 integrons in 5.5% (*n* = 11). All class 2 integrons were only found in the isolates related to the pESI-like positive clone. Class 1 integrons were found in isolates from different animal species: 38.5% (*n* = 10) swine, 23.1% (*n* = 6) chickens, 19.2% (*n* = 5) horses, 11.5% (*n* = 3) cattle, and 7.7% (*n* = 2) turkeys. Among class 1 integrons, we found the *qac*Δ1 and *sul*1 genes at the 3’CS in 23 isolates, the *sul*1 gene only in one isolate, and one isolate was missing the 3′-conserved segment (3’CS). One isolate carried the *qac*L and *sul*3 genes (Supplementary Figure 1).

**Table 2:** Class 1 and Class 2 integrons identified in 26 *Salmonella* Infantis.

ID isolate	Source	Integrase (5’ CS)	IntFinder 1.0				
			Genes	Identity	Query/Template lengh	Integron name	Accesion number
18-006979-231	Swine	*intl*1	*aad*A1	100	1008/1008	In740	JN645874
18-006979-249	Swine	*intl*1	*aad*A1	95.82	1194/1031	In695	AF327727
18-006979-283	Cow	*intl*1	*ere*A2	99.9	1374/1372	Class I	AF326209
18-006979-392	Swine	*intl*1	*aad*A2b	94.05	1010/1009	In128	AF221903
18-012180-313	Swine	*intl*1	*ant*(2″)-la	93.56	767/745	In161	HM367610
18-012180-414	Swine	*intl*1	-	-			
18-024127-087	Chicken	*intl*1	*intl*1, *aad*A1, *qac*Δ1, *sul*1, *orf*5	99.97	3926/3926	In2	AF071413
18-024127-087	Chicken	*intl*2	-	-			
18-024128-077	Horse	*intl*1	*intl*1, *aad*A1, *qac*Δ1, *sul*1, *orf*5	99.97	3926/3926	In2	AF071413
18-024128-077	Horse	*intl*2	*drf*A14, *lsp*	100	2386/2386	In14	EU780012
18-024131-003	Chicken	*intl*1	*intl*1, *aad*A1, *qac*Δ1, *sul*1, *orf*5	99.97	3926/3926	In2	AF071413
18-024131-003	Chicken	*intl*2	*drf*A14, *lsp*	100	2386/2386	In14	EU780012
18-024131-027	Cow	*intl*1	*intl*1, *aad*A1, *qac*Δ1, *sul*1, *orf*5	99.97	3926/3926	In2	AF071413
18-024131-027	Cow	*intl*2	*drf*A14, *lsp*	100	2386/2386	In14	EU780012
18-024131-029	Horse	*intl*1	*intl*1, *aad*A1, *qac*Δ1, *sul*1, *orf*5	99.97	3926/3926	In2	AF071413
18-024131-029	Horse	*intl*2	*drf*A14, *lsp*	100	2386/2386	In14	EU780012
18-038310-060	Horse	*intl*1	*intl*1, *aad*A1, *aac*(3)-*VIa*, *qac*Δ1, *sul*1, *orf*5	99.98	11,129/11129	In1077	CP009409
18-038875-022	Chicken	*intl*1	*intl*1, *aad*A1, *qac*Δ1, *sul*1, *orf*5	99.97	3926/3926	In2	AF071413
18-038875-022	Chicken	*intl*2	-	-			
18-038876-065	Chicken	*intl*1	*intl*1, *aad*A1, *qac*Δ1, *sul*1, *orf*5	99.97	3926/3926	In2	AF071413
18-038876-065	Chicken	*intl*2	*drf*A14, *lsp*	100	2386/2386	In14	EU780012
18-038877-013	Swine	*intl*1	*aad*A2	78.3	1009/1009	ln128	AF221903
18-038877-048	Swine	*intl*1	*intl*1, *ant*(2″)-la, *aad*A2, *qac*Δ1, *sul*1	100	4515/4515	In1071	KJ411925
19-021047-026	Chicken	*intl*1	*intl*1, *aad*A1, *qac*Δ1, *sul*1, *orf*5	99.97	3926/3926	In2	AF071413
19-021047-026	Chicken	*intl*2	*drf*A14, *ls*p	95.48	1084/2386	In14	EU780012
19-021048-003	Swine	*intl*1	-	-			
19-021048-012	Cow	*intl*1	-	-			
19-021048-033	Swine	*intl*1	*intl*1	97.19	55,434/55702	In764	JN983043
19-021048-036	Horse	*intl*1	*intl*1, *aad*A1, *aac*(3)-*VIa*, *qac*Δ1, sul1, orf5	99.98	11,129/11129	In1077	CP009409
19-021048-041	Chicken	*intl*1	*intl*1, *aad*A1, *qac*Δ1, *sul*1, *orf*5	99.97	3926/3926	In2	AF071413
19-021048-041	Chicken	*intl*2	*drf*A14, *lsp*	100	2386/2386	In14	EU780012
19-021048-043	Horse	*intl*1	*intl*1, *aad*A1, *qac*Δ1, *sul*1, *orf*5	99.97	3926/3926	In2	AF071413
19-021048-043	Horse	*intl*2	*drf*A14, *lsp*	100	2386/2386	In14	EU780012
19-021048-045	Turkey	*intl*1	*aad*A8	95.75	847/847	In424	AF326210
19-021048-050	Swine	*intl*1	*intl*1, *aad*A2, *qac*Δ1, *sul*1, *orf*5	99.98	4405/4405	In531	DQ019420
19-021048-057	Turkey	*intl*1	*intl*1, *aad*A1, *qac*Δ1, *sul*1, *orf*5	99.97	3926/3926	In2	AF071413
19-021048-057	Turkey	*intl*2	*drf*A14, *lsp*	100	2386/2386	In14	EU780012

(-) not detected.

In addition to antimicrobial resistance genes, isolates carrying the pESI-like plasmid also carried virulence, heavy metals and biocide resistance genes: *sin*H, *iro*B, *iro*C, *ybt*Q, *ybt*P, *ars*R, *gol*S, *gol*T, *mer*R, *mer*T, *mer*P, *mer*C, and *qac*EΔ1 (Supplementary Figure 1). For the virulence genes, *sin*H encodes an autotransporter protein involved in adhesion and invasion. The *ars*R, *gol*T, and *gol*S are stress response genes, the *ars*R gene confers arsenic tolerance, an essential adaptative capability. The *ybt*Q and *ybt*P virulence genes were found only in isolates carrying the pESI-like plasmid and are associated with iron transport. Within the heavy metal resistance genes, the *mer* operon consisted of the regulatory genes *mer*R and the structural genes *mer*T, *mer*P, and *mer*C, conferring resistance to mercury. Among disinfectants, the *qac*EΔ1 gene confers resistance to ethidium bromide and quaternary ammonium compounds. The 11 pESI-like positive isolates were also positive for *Intl*1 and *Intl*2 genes, which code for the integrases found in class 1 and class 2 integrons, respectively (Supplementary Figure 1). Because all of these AMR gene identifications were made via short-read sequencing on the Illumina® MiSeq®, we cannot confirm if the genes were detected or mediated on a plasmid.

### 3.3. Antimicrobial susceptibility

**Figure 2:**
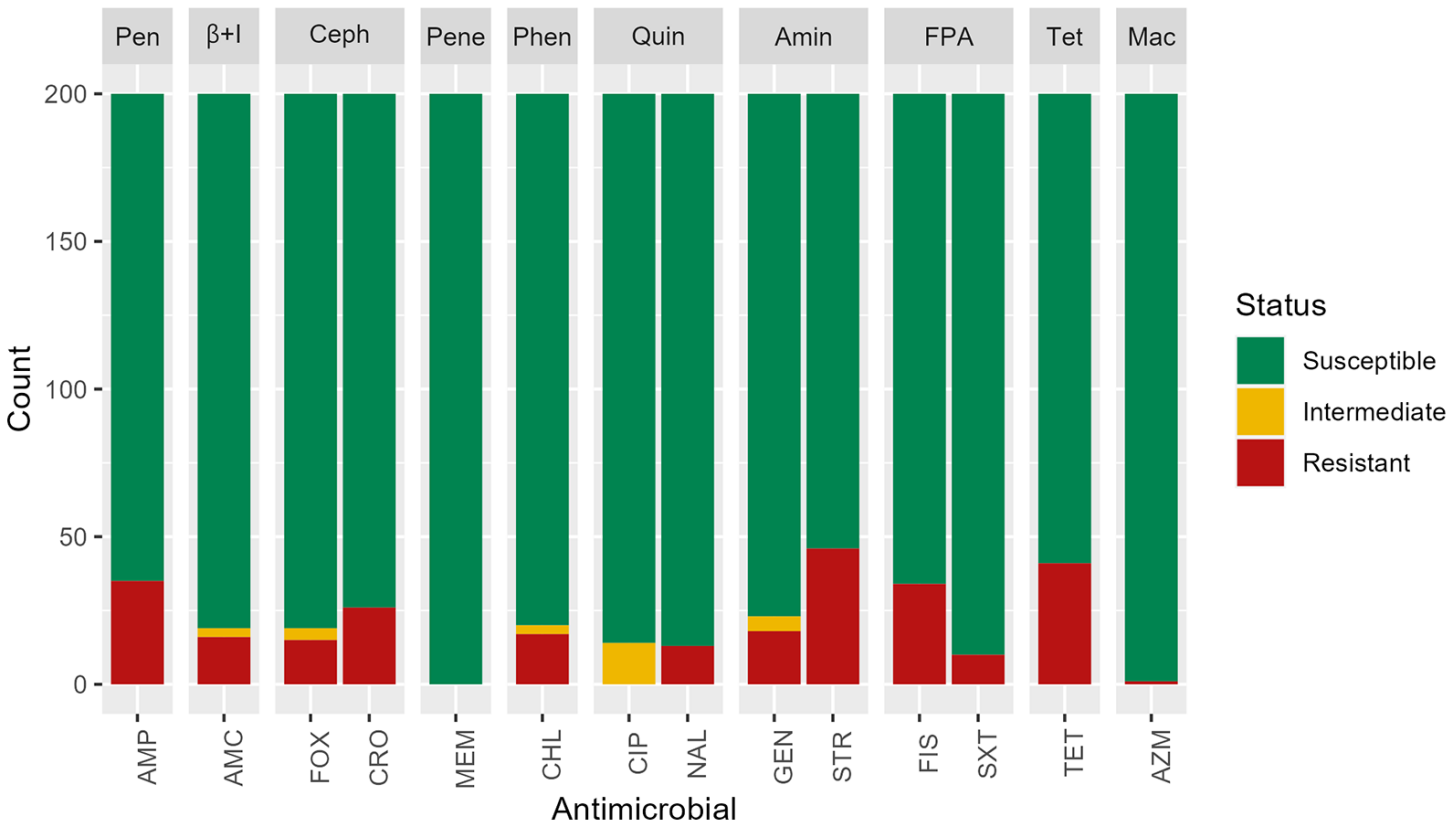
Antimicrobial susceptibility test results of *n* = 200 *Salmonella* Infantis isolates. Antimicrobials were grouped by classes: Pen: penicillins, β + I = β-lactam + β-lactamase inhibitors, Ceph = cephems, Pene = penems, Phen = phenicols, Quin = quinolones, Amin = aminoglycosides, FPA = folate pathway antagonists, Tet = tetracyclines, Mac = macrolides. Antimicrobials: AMP = ampicillin, AMC = amoxicillin/ clavulanic acid, FOX = cefoxitin, MEM = meropenem, CHL = chloramphenicol, CIP = ciprofloxacin, NAL = nalidixic acid, GEN = gentamicin, STR = streptomycin, FIS = sulfisoxazole, SXT = trimethoprim/sulfamethoxazole, TET = tetracycline, AZM = azithromycin.

Based on the phenotypic assays, among the 200 *S. infantis* isolates tested in this study, 33.5% (*n* = 67) had antimicrobial resistance with 11% (*n* = 22) resistant to one antimicrobial, 3% (*n* = 6) resistant to two antimicrobial categories, and 19.5% (*n* = 39) MDR (non-susceptible to more than one antimicrobial in three or more antimicrobial categories). Phenotypic resistance was most common against streptomycin (23%, *n* = 46), tetracycline (20.5%, *n* = 41), ampicillin (17.5%, *n* = 35) and sulfisoxazole (17%, *n* = 34). Resistance to ceftriaxone was found in 13% (*n* = 26) of isolates, most often mediated by *bla*_CMY-2_. All isolates were susceptible to meropenem (Figure 2). Isolates that showed resistance to only one antimicrobial presented resistance to streptomycin (54.5%, *n* = 12), tetracycline (36.4%, *n* = 8), or another antimicrobial (9.1%, *n* = 2).

Among isolates showing intermediate susceptibility to any antimicrobial (14.5%, *n* = 29), 10 did not carry any antimicrobial resistance gene. Five isolates with intermediate susceptibility to gentamicin were positive for the *aac*(3)-IVa gene and one for the *ant*(2″)-Ia gene. Among the 14 isolates with intermediate susceptibility for ciprofloxacin, 13 had a mutation in *gyr*A gene, and one carried the gene *qnr*B19. All isolates presented a chromosomal mutation in *par*C T57S, but the presence of this single mutation did not show reduced susceptibility to ciprofloxacin or resistance to nalidixic acid. A chromosomal mutation in the *gyr*A gene was found in isolates showing resistance to nalidixic acid and decreased susceptibility to ciprofloxacin. A single isolate expressed resistance to macrolides, and three genes (*mph*A, *mph*E, *msr*E) were detected.

**Figure 3:**
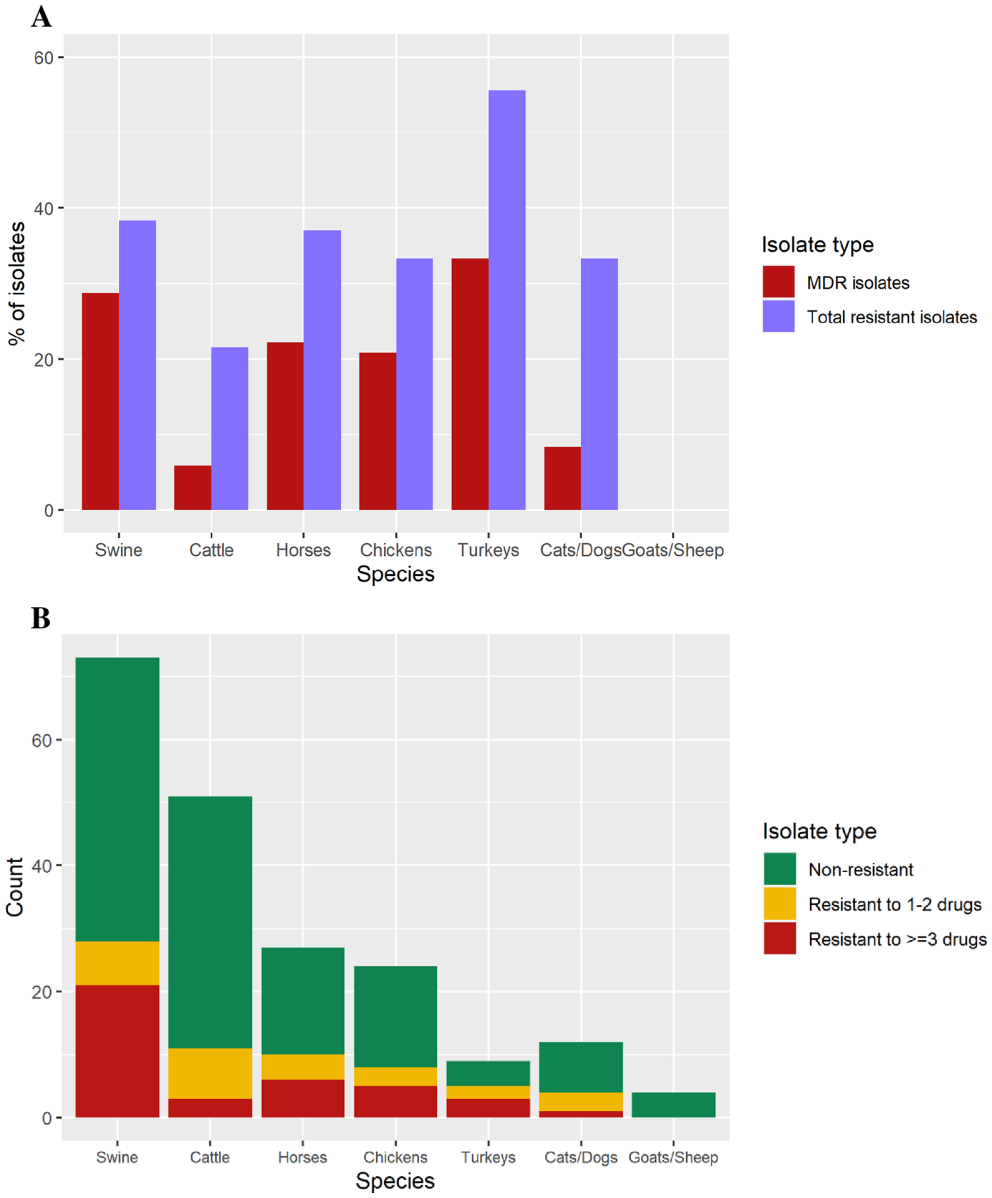
**(A)** Isolates containing antimicrobial resistance, and multi-drug resistance isolates by animal species. **(B)** Number of isolates in each antimicrobial resistance level per species (swine = 74, cattle = 50, horses = 27, chickens = 24, cats/dogs = 12, turkeys = 9, goats = 2, sheep = 2).

The proportion of isolates exhibiting antimicrobial resistance varied by animal species (Figure 3). Turkey isolates showed 55.5% (*n* = 5/9) resistance to at least 1 antibiotic, followed by swine (37.8%, *n* = 28/74), horses (37%, *n* = 10/27), chickens (33.3%, *n* = 8/24), dogs/cats (33.3%, *n* = 4/12), and cattle (22%, *n* = 11/50). Goat and sheep isolates were pan-susceptible (Figure 3).

The 11 MDR diagnostic isolates harboring an IncFIB(K)-1-Kpn3 replicon with several resistance genes, in 10 cases including the ESBL *bla*_CTX-M-65_ gene, also presented a *gyr*A mutation consistent with MDR isolates associated with human cases and the pESI-like megaplasmid. Multidrug resistant isolates were identified from swine (*n* = 21), horses (*n* = 6), chickens (*n* = 5), turkeys (*n* = 3), and cats/dogs (*n* = 1), whereas goat and sheep isolates were pan-susceptible (Table 2). Nine of the 10 *bla*_CTX-M-65_-positive isolates were resistant to ampicillin, ceftriaxone, streptomycin, nalidixic acid, sulfisoxazole, and tetracycline and presented decreased susceptibility to ciprofloxacin. Only one *bla*_CTX-M-65_-positive isolate was susceptible to ampicillin and ceftriaxone (Table 3).

**Table 3:** Resistance phenotypes and genotypes of 11 pESI-like positive isolates in U.S. diagnostic isolates from animals and comparison with one U.S. pESI-like positive isolate from NCBI (SRR5195891, food/retail meat) and the Italian pESI-like strain (ERR1014119, human).

Isolate ID	Source	Date collected	Location	Phenotipic Resistance pattern*	Antimicrobial resistance genes	*gyr*A mutation	Replicon
18-024131-003	Chicken	2016	NC	GEN, STR, CIP, NAL, FIS, SXT, TET	*aac*(3)-IVa, *aad*A1, *aph*(4)-Ia, *sul*1, *dfr*A14, *tet*A	D87Y	IncFIB(K)-1-Kpn3
19-021048-057	Turkey	2017	NC	AMP, CRO, GEN, STR, CHL, CIP, NAL, FIS, SXT, TET	*bla*_CTX-M-65_, *aac*(3)-IVa, *aad*A1, *aph*(4)-Ia, *aph*(3′)-Ia, *flo*R, *sul*1, *dfr*A14, tetA, *fos*A	D87Y	IncFIB(K)-1-Kpn3
18-038875-022	Chicken	2017	MD	AMP, CRO, STR, CHL, CIP, NAL, FIS, TET	*bla*_CTX-M-65_, *aac*(3)-IVa, *aad*A1, *aph*(4)-Ia, *aph*(3′)-Ia, *flo*R, *sul*1, *dfr*A14, *tet*A, *fos*A	D87Y	IncFIB(K)-1-Kpn3
19-021047-026	Chicken	2017	MD	AMP, CRO, GEN, STR, CHL, CIP, NAL, FIS, TET	*bla*_CTX-M-65_, *aac*(3)-IVa, *aad*A1, *aph*(4)-Ia, *aph*(3′)-Ia, *flo*R, *sul*1, *dfr*A14, *tet*A, *fos*A	D87Y	IncFIB(K)-1-Kpn3
18-038876-065	Turkey	2017	NY	AMP, CRO, STR, CHL, CIP, NAL, FIS, SXT, TET	*bla*_CTX-M-65_, *aac*(3)-IVa, *aad*A1, *aph*(4)-Ia, *aph*(3′)-Ia, *flo*R, *sul*1, *dfr*A14, *tet*A, *fos*A	D87Y	IncFIB(K)-1-Kpn3
18-024127-087	Chicken	2016	NY	GEN, STR, CIP, NAL, FIS, TET	*bla*_CTX-M-65_, *aac*(3)-IVa, *aad*A1, *aph*(4)-Ia, *aph*(3′)-Ia, *sul*1, *dfr*A14, *tet*A	D87Y	IncFIB(K)-1-Kpn3
19-021048-041	Chicken	2016	NY	AMP, CRO, GEN, STR, CIP, NAL, FIS, SXT, TET	*bla*_CTX-M-65_, *aac*(3)-Iva, *aad*A1, *aph*(4)-Ia, *aph*(3′)-Ia, *sul*1, *dfr*A14, *tet*A	D87Y	IncFIB(K)-1-Kpn3
19-021048-043	Horse	2016	NY	AMP, CRO, GEN, STR, CHL, CIP, NAL, FIS, SXT, TET	*bla*_CTX-M-65_, *aac*(3)-IVa, *aad*A1, *aph*(4)-Ia, *aph*(3′)-Ia, *flo*R, *sul*1, *dfr*A14, *tet*A, *fos*A	D87Y	IncFIB(K)-1-Kpn3
18-024131-029	Horse	2016	NY	AMP, CRO, STR, CHL, CIP, NAL, FIS, SXT, TET	*bla*_CTX-M-65_, *aac*(3)-IVa, *aad*A1, *aph*(4)-Ia, *aph*(3′)-Ia, *flo*R, *sul*1, *dfr*A14, *tet*A, *fos*A	D87Y	IncFIB(K)-1-Kpn3
18-024128-077	Horse	2016	NY	AMP, CRO, STR, CHL, CIP, NAL, FIS, SXT, TET	*bla*_CTX-M-65_, *aac*(3)-IVa, *aad*A1, *aph*(4)-Ia, *aph*(3′)-Ia, *flo*R, *sul*1, *dfr*A14, *tet*A, *fos*A	D87Y	IncFIB(K)-1-Kpn3
18-024131-027	Cow	2016	DE	AMP, CRO, GEN, STR, CHL, CIP, NAL, FIS, SXT, TET	*bla*_CTX-M-65_, *aac*(3)-IVa, *aad*A1, *aph*(4)-Ia, *aph*(3′)-Ia, *flo*R, *sul*1, *dfr*A14, *tet*A	D87Y	IncFIB(K)-1-Kpn3
SRR5195891	Chicken	2016	DE	ND	*bla*_CTX-M-65_, *aac*(3)-IVa, *aad*A1, *aph*(4)-Ia, *aph*(3′)-Ia, *flo*R, *sul*1, *dfr*A14, *tet*A, *fos*A	D87Y	IncFIB(K)-1-Kpn3
ERR1014119	Human [6]	2014	ITALY	AMP, CTX, GEN, KAN, CHL, CIP, NAL, SMX, TMP, TET	*bla*_CTX-M-65_, *aac*(3)-IVa, *aad*A1, *aph*(4)-Ia, *aph*(3′)-Ic, *flo*R, *sul*1, *dfr*A14, *tet*A, *fos*A	ND	IncP

* AMP: ampicillin, CRO: ceftriaxone, STR: streptomycin, CHL: chloramphenicol, CIP: ciprofloxacin, NAL: nalidixic acid, FIS: sulfisoxazole, SXT: trimethoprim/sulfamethoxazole, SMX: sulfamethoxazole, TMP: trimethoprim, TET: tetracycline, ND: not determined.

The 11 isolates carrying the pESI-like plasmid, also had a *gyr*A mutation (D87Y) and 4 additional antimicrobial resistance (AMR) genes: *aph*(4)-Ia (100%), *aac*(3)-VI (100%), *sul*1 (100%), and *tet*A (100%). Often, these isolates also contained the *bla*_CTX-M-65_ (90.9%), *aph*(3)-Ia (90.9%), *dfr*A14 (90.9%), *flo*R (72.7%), and *fos*A (63.6%) genes. Comparing these 11 isolates with U.S. pESI-like positive strains published in the NCBI database, and with the published MDR ESBL-producing *S. infantis* from a clonal lineage in Italy, we observed that these 11 isolates were related to the pESI-like clone (Supplementary Figure 1).

All isolates carrying the *bla*_CMX-M-65_ gene (*n* = 10) harbored the plasmid IncFIB(K)-1-kpn3. All *bla*_CTX-M-65_ positive isolates were MDR, also carrying the following antimicrobial resistance genes: *aac*(3)-IVa, *aad*A1, *aph*(3′)-Ia, *aph*(4)-Ia, *sul*1, and *tet*A. Nine of ten also carried the *dfr*A14 genes, 8 of 10 the *flo*R gene, and 7 of 10 the *fos*A gene (Table 3).

### 3.4. Chronicity and changes of the isolates over the study timeframe

We identified 12 isolates with extended spectrum β-lactamase (ESBLs) genes. The ESBL *bla*_SHV-12_ was only found in 2 isolates from swine only during 2014, whereas the *bla*_CTX-M-65_ gene was found in 10 isolates from multiple species: chickens (*n* = 4), horses (*n* = 3), turkeys (*n* = 2) and cattle (*n* = 1) only during the years 2016 and 2017 (Figure 1).

**Figure 4:**
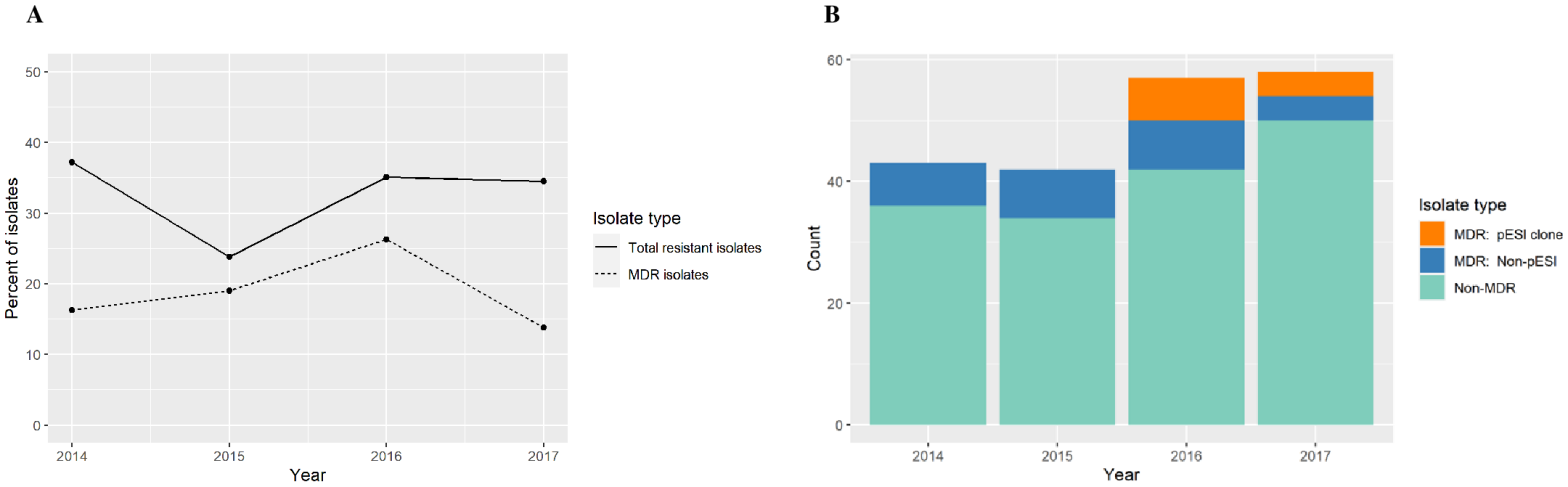
**(A)** Antimicrobial resistance annual trend among *S. infantis* isolates from different animal species. **(B)** Count data for multi-drug resistant (MDR) isolates, MDR *S. infantis* isolates harboring a pESI-like megaplasmid, and non-MDR isolates.

The antimicrobial resistance annual trend showed little variation through the years (Figure 4A). After a logistic regression analysis, we conclude that evidence is not strong enough to reject the null hypothesis that the odds ratio (and thus the proportion of isolates with resistance) is independent of year (*p* = 0.53). However, this finding may be an artifact of including only 4 years’ data in the analysis (Figure 4A).

Among MDR isolates, we found that 46.7% of isolates in 2016 and 50% of isolates in 2017 were of the emergent *S. infantis* clone (Figure 4B). In our study, the pESI-like clone have not been detected prior to 2016. In addition, the isolates studied were submitted for diagnostic or surveillance purposes depending of the need of the submitter and in those submissions we captured the emergence of a new strain.

## 4. Discussion

This study captured the emergence of an MDR *S. infantis* clone in animals harboring a pESI-like megaplasmid containing an ESBL *bla*_CTX-M-65_ gene.

*S. infantis* is commonly isolated from food animals in the U.S.; it has been found in chickens, turkeys, cattle, and swine (Tate et al., 2017). Although antimicrobial resistance in *S. infantis* is historically lower than other *Salmonella* serotypes in poultry products, an MDR pESI-like positive clone has emerged associated with poultry products and became widespread by 2019 in U.S. poultry (Tyson et al., 2021).

In this study, we found that 19.5% of the *S. infantis* isolates were MDR, with a higher prevalence of resistance in turkeys (33.3%), swine (28.8%), horses (22.2%), and chickens (20.8%). The most common antimicrobial resistance among isolates was against streptomycin (23%), tetracycline (20.5%), ampicillin (17.5%), and sulfisoxazole (17%). All *S. infantis* isolates tested presented a chromosomal mutation in *par*C T57S. This mutation has been associated with reduced susceptibility in other non-typhoidal *Salmonella* (Ling et al., 2003). In the *S. infantis* isolates studied, the presence of this single mutation did not show reduced susceptibility to ciprofloxacin or resistance to nalidixic acid. Our findings are in concordance with a *Salmonella* study that demonstrated this mutation does not confer reduced susceptibility when present with no additional mutations in the DNA gyrase or topoisomerase genes (Jovčić et al., 2020). In our study, isolates showing the additional presence of a *gyr*A mutation displayed resistance to nalidixic acid and decreased susceptibility to ciprofloxacin.

Among aminoglycoside resistance genes, all isolates were positive for *aac*(6′)-laa, a gene that confers resistance to tobramycin, kanamycin and amikacin (antimicrobials not tested). This gene seems to have no clinical and/or evolutionary significance (Biskri and Mazel, 2003). Resistance to the aminoglycosides tested was associated with the aminoglycoside modifying enzymes *aad*A1 (streptomycin), and *aac*(3)-IVa (gentamicin). Resistance to tetracycline was associated with the efflux transporter *tet*A gene, sulfonamide with the *sul*1 gene, and chloramphenicol with the *flo*R gene. Phenotypic resistance to azithromycin (a macrolide) was found in only one isolate that carried the *mph*A, *mph*E, and *msr*E genes. Brown et al. (2018) studied the macrolide resistance phenotype conferred by two different *ere*A alleles and found each allele produced the same phenotypic antimicrobial resistance pattern, that of conferring resistance to erythromycin and clarithromycin while retaining antimicrobial activity for azithromycin or telithromycin. The five isolates in this study with the *ere*A gene did not show resistance to azithromycin, but erythromycin or clarithromycin were not tested.

Resistance against β-lactams was associated with the presence of *bla*_CMY-2_, *bla*_TEM-1_, *bla*_CTX-M-65_ and *bla*_SHV-12_ genes, the last two coding for ESBLs. The *bla*_SHV-12_ gene was found in 2 isolates from swine in 2014, and the *bla*_CTX-M-65_ gene was found in 10 isolates from chickens (*n* = 4), horses (*n* = 3), turkeys (*n* = 2), and cattle (*n* = 1) during the years 2016 and 2017; and all 10 isolates were related to the ESI clone. Poultry seem to be the main source of the *bla*_CTX-M_ gene in *S. infantis* infections. Franco et al. (2015) demonstrated the transmission from broiler chickens and chicken meat to humans in Italy. Retail chicken meat was also linked to human infections in the U.S. (Hindermann et al., 2017). Tate et al. (2017) found *S. infantis* related to the ESI clone that had emerged in Europe harboring a *bla*_CTX-M-65_ gene in retail chicken meat, humans, and food animals during 2013–2015 (Aviv et al., 2014; Franco et al., 2015). This strain was also found in Switzerland from poultry meat and human infections from isolates recovered during 2010–2015 (Nagy et al., 2020). It was demonstrated that the earliest case of CTX-M-65 β-lactamase *S. infantis* infection in the U.S. was among travellers from South America and subsequent infections happened within the country (Tate et al., 2017). It is not possible to determine when or how this strain was introduced into poultry in the United States, but it could be a consequence of the international distribution of infected breeder stocks, chicken feed, or feed additives contaminated with the pESI-like strain (Nagy et al., 2020). U.S. MDR pESI-like positive isolates show resistance to many antibiotics and the presence of CTX-M β-lactamases in this strain is concerning due to the expression of ESBLs that confer resistance to ceftriaxone and ampicillin, two recommended drugs for the treatment of salmonellosis (Hindermann et al., 2017). Although the *bla*_CTX-M_ gene is not always present in the phenotypic antimicrobial resistance pattern of ESI strains (Burnett et al., 2021), the megaplasmid carried by the ESI clone harbors additional antimicrobial resistance genes; therefore, an infection caused by this ESBL-positive strain may be difficult to treat with commonly used antibiotics. In Italy, Franco et al. (2015) studied ESBL-positive *S. infantis* isolates from broilers, broiler meat and human cases. They found that most isolates carried the *bla*_CTX-M-1_ ESBL and one human isolate carried a *bla*_CTX-M-65_ ESBL. We compared the human isolate with the *bla*_CTX-M-65_ ESBL to our isolates (shown in Table 2) and observed it to have a closer genetic relatedness to Group 1, containing all pESI-like positive isolates (Figure 1).

In China, a study found that most *Salmonella* isolates that harbored the IncFIB(K)-1-Kpn3 plasmid replicon were multidrug-resistant (Liu et al., 2020). They also observed that IncFIB(K)-1-Kpn3-positive strains carry more antimicrobial resistance than isolates with other replicon types. In our study, 46.7% of isolates in 2016 and 50% of isolates in 2017 carrying the IncFIB(K)-1-Kpn3 plasmid (pESI-like) were also MDR; and all were related to the emergent *S. infantis* clone.

The pESI-like positive clone described in the U.S. has a large megaplasmid with several resistance and virulence genes and a *gyr*A chromosomal mutation conferring fluoroquinolone resistance (Tyson et al., 2021). The combination of resistance genes varies among the isolates, but they always comprise different combinations of a set of 10 resistance genes: *tet*A, *sul*1, *aad*A1, *aac*(3)-IV, *aph*(4)-la, *flo*R, *dfr*A14, *aph*(3′)-la, *fos*A3, and the extended-spectrum beta-lactamase gene *bla*_CTX-M-65_ (Tyson et al., 2021). Two different pESI-like isolates with ESBL genes were observed: one type carrying the *bla*_CTX-M-1_ or *bla*_CTX-M-65_ gene in Europe (Franco et al., 2015) and another carrying the *bla*_CTX-M-65_ gene in American isolates (Alba et al., 2020). We also detected pESI-like positive isolates carrying the *bla*_CTX-M-65_ gene.

It was reported that the combination of the resistance genes among isolates from animals was more consistent than in retail meat (Franco). In our study, all isolates related to the Italian ESI clone showed the presence of 5 genes [*aac*(3)-IVa, *aad*A1, *aph*(4)-Ia, *sul*1, *tet*A], a mutation in *gyr*A D87Y, and an IncFIB-like plasmid. Tyson et al. (2021) found that 40.7% of retail meat and 72.6% of human isolates with the pESI megaplasmid also carried the *bla*_CTX-M-65_ gene. In our study, 90.9% of isolates related to the ESI clone carried this ESBL *bla*_CTX-M-65_ gene. The emergent MDR megaplasmid in *S. infantis* strains has been found in different replicons. It was found in an IncFIB-like replicon in several countries in the Americas: U.S. (Tate et al., 2017; Nagy et al., 2020; Tyson et al., 2021), Ecuador (Mejía et al., 2020), Galapagos (Burnett et al., 2021); in an IncFII replicon in Switzerland (Nagy et al., 2020), and in an IncP/IncI chimeric replicon in Israel (Aviv et al., 2014) and Italy (Franco et al., 2015).

Franco et al. (2015) suggested that the acquisition of the *bla*_CTX-M-1_ gene in the IncP replicon in the Italian ESI isolates could be from commensal *E. coli* population of the intestinal microbiota of broiler chickens. The IncFIB-like plasmid in the U.S. pESI-like positive isolates seems to have emerged from multiple recombination events (Tyson et al., 2021). In the Netherlands, the IncFIB replicon has been detected in *E. coli* isolated from poultry carrying CTX-M ESBLs (Dierkx et al., 2010).

Carriage of the pESI-like megaplasmid in U.S. strains appears to be limited to *S. infantis* (Dierkx et al., 2010). McMillan et al. (2020) observed a carriage rate increase from 2017 to 2018. Tyson et al. (2021) showed that the prevalence of pESI-like positive isolates among retail meat increased from 6.7% in 2014 to 29.2% in 2019. In this study, we found the pESI-like megaplasmid in 11 isolates from animals in 2016 and 2017.

According to Aviv et al. (2014), ESI is characterized by adaptive chromosomal mutations and the presence of a megaplasmid, which confers resistance to multiple drugs, heavy metals, and disinfectants. All isolates in our study showed a *gyr*A chromosomal mutation, several combinations of 10 resistance genes, virulence genes (*sin*H, *iro*B, *iro*C, *ybt*Q, and *ybt*P), heavy metals genes (*ars*R, *gol*S, *gol*T, *mer*R, *mer*T, *mer*P, and *mer*C), and a biocide gene (*qac*Δ1). Virulence genes are essential for the level of iron required for *Salmonella* survival and help to increase its pathogenicity. The same applies to the heavy metal *ars*R gene which is necessary for arsenic tolerance and is present in successful epidemic clones of *Salmonella* (Guerra et al., 2000). The *ybt* genes code for yersiniabactin, a siderophore-dependent iron uptake system, found only in the pESI-like positive strains, and is a characteristic of the pESI clone (Aviv et al., 2014).

Integrons are associated with the global spread of antimicrobial resistance (Kaushik et al., 2018). Class 1 integrons are the most commonly reported integrons in MDR *Salmonella* strains (Guerra et al., 2000; Askari Badouei et al., 2021; U.S. Food and Drug Administration, 2021). In a study carried out in Iran (Kaushik et al., 2018) with *S. infantis* isolated from broiler farms, class 1 integrons were detected in 95.6% of MDR *S. infantis*, but no class 2 integrons were detected. A study carried out in Spain with MDR *S. infantis* isolated from pigs found class 1 (77.4%) and class 2 (6.5%) integrons, with class 1 integrons being the most prevalent in MDR *Salmonella* isolated from swine (Argüello et al., 2018). In the present study, we found class 1 integrons in 66.7% of MDR isolates and class 2 integrons in 28.2% of MDR *S. infantis*, with the class 2 integrons being only found in the pESI-like positive isolates: chickens (*n* = 2), horses (*n* = 3), turkeys (*n* = 2), and cattle (*n* = 1). Class 1 integrons were found in isolates from different animal species: swine (*n* = 10), chickens (*n* = 6), horses (*n* = 5), cattle (*n* = 3), and turkeys (*n* = 2). The presence of *aad*A1, *sul*1, *drf*A and *qac*Δ1 genes indicate the presence of a class 1 integron (Li et al., 2021) with *qac*Δ1 and *sul*1 genes in the constant region and several resistant genes in the variable region (Orman et al., 2002). Using the IntFinder Tool, we could detect the *aad*A1 gene in 57.7% of the class 1 integrons and the *drf*A gene in 81.8% of the class 2 integrons. Our results differed from Aviv et al. (2014), where they found a class I integron in pESI-like positive isolates encoding the gene *dfr*A1.

All isolates in our study harbored the *mds*A/B genes encoding a membrane fusion protein of the multidrug and metal efflux complex MdsABC, the virulence genes *sin*H, *iro*B and *iro*C, and the heavy metal resistance genes *ars*R, *gol*S, and *gol*T. The *mds*A/B, *gol*T, *gol*S, and *sin*H genes were observed in over 90% of non-typhoidal *Salmonella* spp. circulating among animals and animal products in South Africa from 1957 to 2019 (Carroll et al., 2021).

In our study, 99% of the isolates were classified as ST32. In accordance with other studies, *S. infantis* is usually ST32; and the globally disseminated pESI-like clone belongs to ST32 (Alba et al., 2020; Liu et al., 2020; Burnett et al., 2021) with locus variants of ST32 (Mejía et al., 2020). Among Group 1 containing ESI related isolates, we found a separation of 7 to 27 SNPs among the animal isolates tested and isolates related to the pESI-like clone from other sources. These findings are in accordance with a previous study involving isolates from humans, food animals and retail chicken meat in the U.S. (Tate et al., 2017).

## 5. Conclusion

In this study, we described the antimicrobial resistance and genomics of *S. infantis* isolated from a variety of animal species in the U.S. during 2014–2017. In this dataset we captured the emergence of an MDR clone in 2016 in multiple animal species that is genetically similar to the pESI-like clone that emerged in Europe. U.S. pESI-like positive isolates harbor several antimicrobial resistance, heavy metal and virulence genes and usually harbor an ESBL gene that confers resistance to ceftriaxone, a third-generation cephalosporin that is used to treat invasive salmonellosis.

Using whole genome sequencing, we found 11 isolates related to the ESI clone in 2016 and 2017, nine of them carrying the ESBL *bla*_CTX-M-65_ gene. We could observe the role of an IncFIB-like megaplasmid in the dissemination of an MDR *S. infantis* clone a with similar AMR pattern through poultry, cattle, and horses.

## Data availability statement

The datasets presented in this study can be found in online repositories. The names of the repository/repositories and accession number(s) can be found at: https://www.ncbi.nlm.nih.gov/genbank/, PRJNA789479.

## Author contributions

MS and LS: conceptualization. MS and JH: methodology, formal analysis, and data curation. JH: software validation. MS and BM-S: investigation. LS: resources, supervision, project administration, and funding acquisition. CT: statistics. MS: writing – original draft preparation. MS, BM-S. JH, CT, TM, and LS: writing – review and editing. MS and CT: visualization. All authors contributed to the article and approved the submitted version.

## Funding

This research was funded by an appointment to the Research Participation Program at the Animal and Plant Health Inspection Service, United States Department of Agriculture, administered by the Oak Ridge Institute for Science and Education through an interagency agreement between the U.S. Department of Energy and USDA APHIS.

## Conflict of interest

The authors declare that the research was conducted in the absence of any commercial or financial relationships that could be construed as a potential conflict of interest.

## Publisher’s note

All claims expressed in this article are solely those of the authors and do not necessarily represent those of their affiliated organizations, or those of the publisher, the editors and the reviewers. Any product that may be evaluated in this article, or claim that may be made by its manufacturer, is not guaranteed or endorsed by the publisher.

## Footnotes

^1^ https://github.com/USDA-VS/vSNP
