# Supplementary material for: Whole-genome sequencing and phylogenetic analysis capture the emergence of a multi-drug resistant Salmonella enterica serovar Infantis clone from diagnostic animal samples in the United States
Source: Front Microbiol. 2023 Jun 2;14:1166908. doi: 10.3389/fmicb.2023.1166908 (PMC10272548; doi:10.3389/fmicb.2023.1166908)
Supplement: Supplementary file 1 [file Data_Sheet_1.docx]

Supplementary Material

Whole-genome sequencing and phylogenetic analysis capture the emergence of a multi-drug resistant *Salmonella enterica* serovar Infantis clone from diagnostic animal samples in the United States

Mariela E Srednik^1*^, Brenda R Morningstar-Shaw^1*^, Jessica A Hicks^1^, Christopher Tong^2^, Tonya A Mackie^1^, Linda K Schlater^1^

*** Correspondence:** Corresponding Author: mariela.srednik@gmail.com

# Supplementary Data

**Supplementary Table 1.** Description (sample of origin, year, carriage of genes) of the 14 U.S. *S.* Infantis positive isolates selected from NCBI.

| **Isolate ID** | **Source** | **Date collected** | **Site** | **Resistance genes** | **GyrA mutation** | **Plasmid** |
| --- | --- | --- | --- | --- | --- | --- |
| SRR2939568 | comminuted chicken | 2014 | NC | aac(3)-Iva, aadA1, aph(4)-Ia, aph(3')-Ia, sul1, dfrA14, tetA | D87Y | IncFIB(K)-1-Kpn3 |
| SRR13694425 | chicken breast | 2020 | CA | aac(3)-Iva, aadA1, aph(4)-Ia, floR, sul1, dfrA14, tetA | D87Y | IncFIB(K)-1-Kpn3 |
| SRR14551805 | chicken carcass | 2021 | SC | aadA1, sul1, tetA | D87Y | IncFIB(K)-1-Kpn3 |
| SRR7815483 | comminuted chicken | 2018 | GA | aadA1, sul1, tetA | D87Y | IncFIB(K)-1-Kpn3, ColpVC |
| SRR7822686 | comminuted turkey | 2018 | NC | aadA1, floR, sul1, dfrA14, tetA | D87Y | IncFIB(K)-1-Kpn3, Col(MG828) |
| SRR8575574 | missing (CDC, host-associated) | 2019 |  | blaCTX-M-65, aac(3)-Iva, aadA1, aph(4)-Ia, aph(3')-Ia, floR, sul1, dfrA14, tetA, fosA | D87Y | IncFIB(K)-1-Kpn3 |
| SRR5508312 | missing (CDC, host-associated) | 2017 |  | blaCTX-M-65, aac(3)-Iva, aadA1, aph(4)-Ia, aph(3')-Ia, floR, sul1, dfrA14, tetA | D87Y | IncFIB(K)-1-Kpn3 |
| SRR5195891 | raw chicken | 2016 | DE | blaCTX-M-65, aac(3)-Iva, aadA1, aph(4)-Ia, aph(3')-Ia, floR, sul1, dfrA14, tetA, fosA | D87Y | IncFIB(K)-1-Kpn3 |
| SRR6976762 | comminuted chicken, | 2018 | NJ | aac(3)-Iva, aadA1, aph(4)-Ia, floR, sul1, dfrA14, tetA | D87Y | IncFIB(K)-1-Kpn3 |
| SRR10392526 | missing (CDC, host-associated) | missing |  | blaCTX-M-65, aac(3)-Iva, aadA1, aph(4)-Ia, aph(3')-Ia, floR, sul1, dfrA14, tetA | D87Y | IncFIB(K)-1-Kpn3 |
| SRR13360718 | chicken breast | 2020 | PA | blaCTX-M-65, aac(3)-Iva, aph(4)-Ia, aph(3')-Ia, floR | D87Y | IncFIB(K)-1-Kpn3 |
| SRR10667035 | turkey pattie | 2019 | MO | aph(3')-Ia, sul1, dfrA14, tetA | D87Y | IncFIB(K)-1-Kpn3 |
| SRR13924096 | missing (CDC, host-associated) | 2021 |  | aac(3)-Iva, aadA1, aph(4)-Ia, aph(3')-Ia, sul1, tetA | D87Y | IncFIB(K)-1-Kpn3 |
| SRR11681148 | comminuted chicken | 2020 | CA | aadA1, floR, sul1, dfrA14, tetA | D87Y | IncFIB(K)-1-Kpn3 |

**
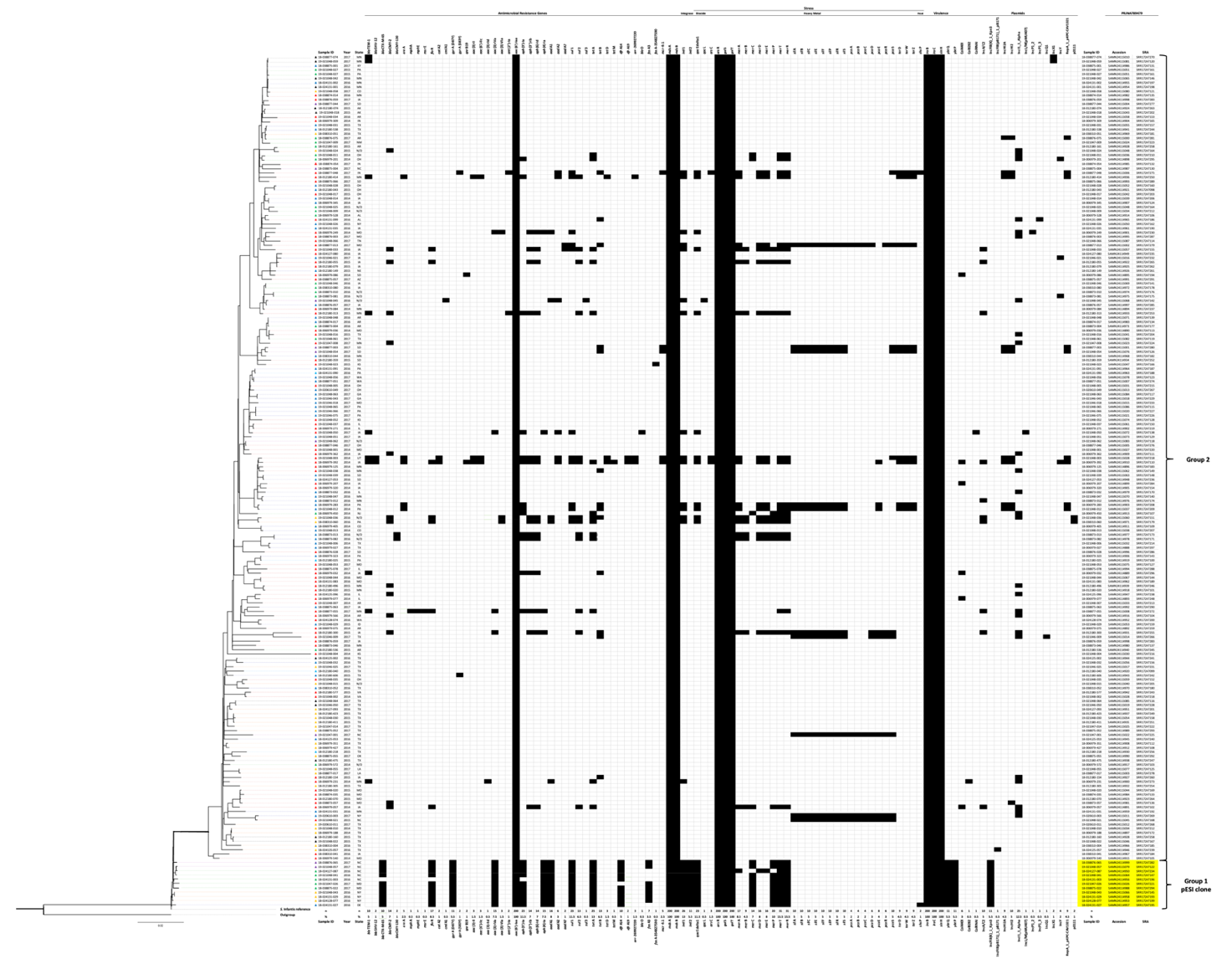
**

**Supplementary Figure 1.** *Salmonella* Infantis phylogenetic tree. Different animal species are in colored triangles: swine (red), cattle (blue), horses (orange), goats/sheep (light blue), chickens (green), turkeys (purple), dogs/cats (black). Group 1 shows pESI-like positive isolates and Group 2 shows different clades of *S*. Infantis isolated from different animal species.

(This Complementary Figure 1 was also uploaded as a PDF file for high-resolution)
