# Supplementary figures and images for: Whole-genome sequencing and phylogenetic analysis capture the emergence of a multi-drug resistant Salmonella enterica serovar Infantis clone from diagnostic animal samples in the United States
Source: Front Microbiol. 2023 Jun 2;14:1166908. doi: 10.3389/fmicb.2023.1166908 (PMC10272548; doi:10.3389/fmicb.2023.1166908)

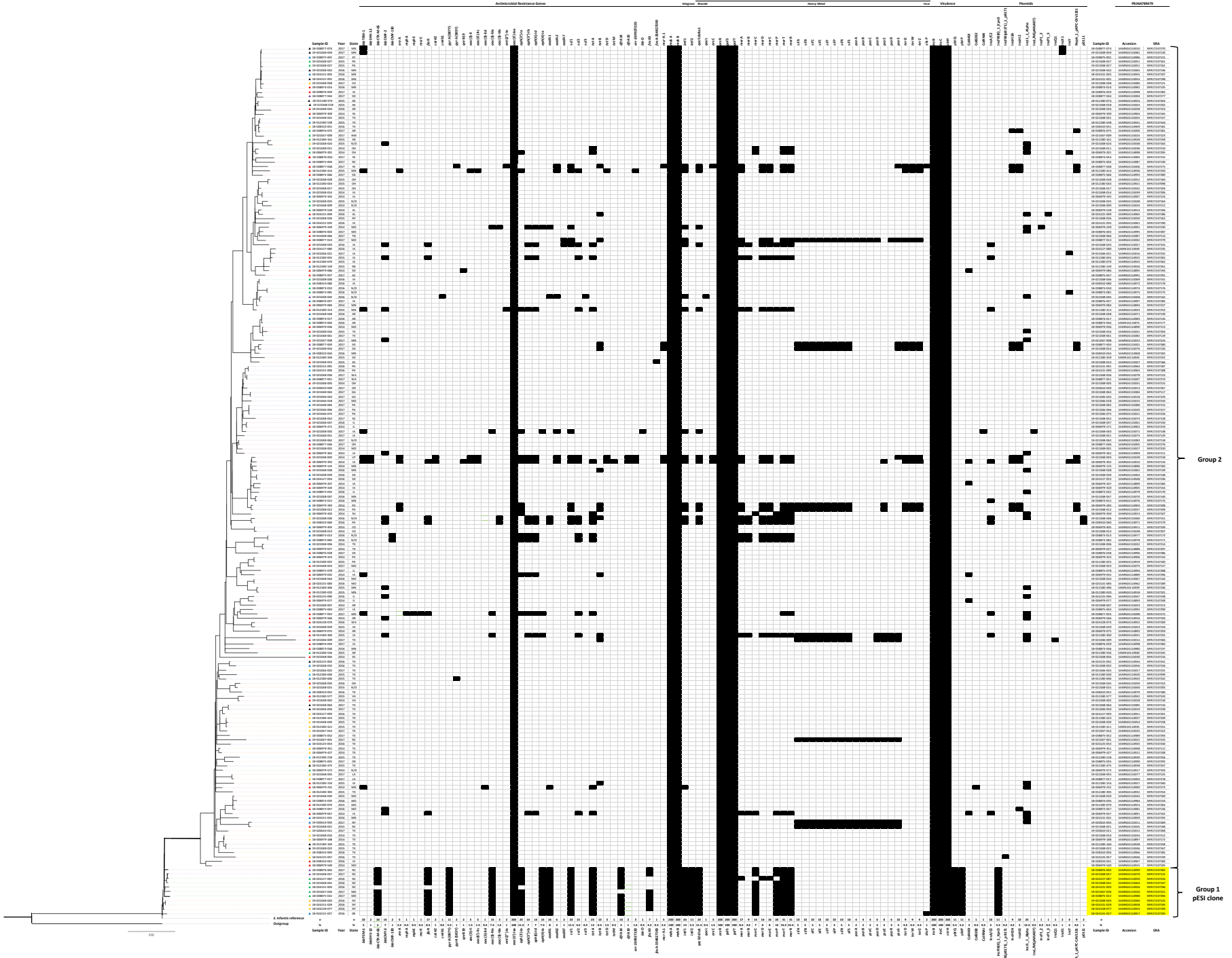

Group 2

Group 1  
pE1 clone

Supplement: Supplementary file 2 [file Data_Sheet_2.pdf]
